# Low-temperature green synthesis of few-layered graphene sheets from pomegranate peels for supercapacitor applications

**DOI:** 10.1038/s41598-023-42029-w

**Published:** 2023-09-20

**Authors:** Prince Anagbonu, Mohsen Ghali, Ahmed Allam

**Affiliations:** 1 Basic and Applied Sciences Institute, Egypt-Japan University of Science and Technology, New Borg El-Arab, 21934 Alexandria Egypt; 2 Physics Department, Faculty of Science, Kafrelsheikh University, Kafrelsheikh, Egypt; 3 Department of Electronics and Communications Engineering, Faculty of Engineering, Egypt-Japan University of Science and Technology, New Borg El-Arab, 21934 Alexandria Egypt

**Keywords:** Energy science and technology, Engineering, Materials science, Nanoscience and technology, Physics

## Abstract

Graphene presents practical applications in energy storage devices, especially supercapacitors. However, mainstream synthesis of graphene includes toxic chemical usage, which threatens the environment. With the recent attention shift to synthesizing nanomaterials from agro-waste due to their easy availability, cost-effectiveness, and, most importantly, their environmental friendliness, we present, in this work for the first time, a novel and green synthesis of few-layered graphene sheets using pomegranate peels as a precursor at a low temperature of 80 °C. The surface morphology and microstructural properties are determined by Transmission Electron Microscopy (TEM), Energy Dispersive X-Ray spectroscopy (EDX), X-Ray Diffraction (XRD), Fourier Transform Infrared Spectroscopy (FTIR), UV–visible spectroscopy (UV–vis), and the electrical properties determined by Hall Effect Measurement. The application as a supercapacitor is also examined using Cyclic Voltammetry (CV), Charge–Discharge Cycling (GCD), and Electrochemical Impedance Spectroscopy (EIS). The resulting supercapacitor delivers an areal capacitance of $3.39 mF{cm}^{-2}$ at a current density of 15.6 μA ${cm}^{-2}$, making our synthesized graphene a good choice for electrochemical storage devices.

## Introduction

Graphene is a two-dimensional planar structure of s${p}^{2}$ hybridized carbon atoms^1^ arranged in a honeycomb crystal lattice^2^ with an inter-layer spacing of $\sim$ 3.35 Å. Graphite crystals, nanotubes, and fullerenes can be formed from graphene by stacking layers, rolling in a specific direction, or wrapping them into a ball^3^. Graphene is a unique nanomaterial with zero band gap^4^ and the only carbon allotrope where carbon atoms are tightly bonded to their neighbors by a unique electronic cloud deviating slightly from quantum mechanical principles^5^. These electrons move freely between the 2pz orbitals^1^, explaining why graphene exhibits unparalleled electrical conductivity^5^.

Graphene exhibits excellent electronic, mechanical, and thermal properties^6^. Graphene’s unique large surface area finds practical applications in energy storage, specifically batteries and supercapacitors^7^. Despite all these excellent properties and uses, the synthesis of high-quality, large-area graphene in a cost-effective way remains a significant challenge. Graphene was first isolated through mechanical exfoliation^8^, which has remained the typical conventional method of graphene synthesis^8^. Also, synthesis has been carried out via routes such as chemical exfoliation, chemical synthesis, thermal chemical vapor deposition, and unzipping carbon nanotubes^9^.

A significant drawback to the conventional graphene synthesis method involves using chemicals that are usually highly corrosive, explosive, and toxic^10^. The process’s cost of highly pure graphite remains another challenge^11^. Consequently, there is an attention shift to the synthesis of nanomaterials and graphene from natural sources^12^, most especially agro-waste^13,14^. Apart from easy availability and access to agro-waste, synthesis from agro-waste is straightforward, safe, and typically produces more stable materials^15^. They also offer an effective way of waste management^13,16^. Consequently, there has been the synthesis of nanomaterials and graphene from waste in general and agro-waste as well^17^^,^^18^ and used in electrochemical storage devices, wastewater treatment, and metal ion sensing, among others^10^. For example, graphene nanosheets were obtained by oxidizing graphite into graphene oxide using the Hummer method and highly concentrated acids, then using pomegranate juice as a reducing and capping agent to reduce graphene oxide^19^. Also, rice husk was combusted at 1123 K and chemically treated to obtain graphene sheets^20^. Furthermore, green tea-synthesized graphene sheets using a high-temperature pyrolysis technique have also been reported^21^. In all these works, graphene was obtained at elevated temperatures, and the synthesis process includes several steps, including chemical treatment.

Pomegranate peels constitute about 43% w/w of the entire fruit^22^ and are most readily available as waste, globally estimated to be 1.62 million tons^22^. The pomegranate peel contains a variety of bioactive substances, including hydrolyzable tannins, flavonoids, complex polysaccharides, and minerals, with gallic acid and punicalagin being the major phenolic compounds which makes them useful in many fields^23^. Although the peels have been utilized in synthesizing carbon dots for several applications^24–28^, the low-temperature synthesis of graphene from the pomegranate peel is yet to be reported.

Herein, we report on a one-pot synthesis of few-layered graphene sheets from a green and abundant precursor of pomegranate peel which was used solely to produce graphene, where no industrial chemicals were used in the synthesis process. The latter makes our method entirely green and energy efficient as the synthesis temperature does not exceed 80 °C. The morphology, microstructure, and electrical properties are determined, and the potential application as a supercapacitor is also examined.

## Materials and methods

### Sample preparation

Pomegranate fruit was sourced from a local market. The peel was removed and washed thoroughly with distilled water to remove the dust and impurities. The clean peels were subsequently dried in an oven at 40 °C overnight and blended into powder form using a blender.

### Synthesis of few-layered graphene sheets

The few-layered graphene sheets were synthesized via the hydrothermal method as follows:

A mass of 0.3 g of fine pomegranate peel powder was dissolved in 45 ml of distilled water and sonicated for 60 min. The solution was then transferred into a 750 ml Teflon-Line stainless steel autoclave and heated at 80 °C for 10 h. The solution was allowed to cool naturally, and filtration using a syringe and a 0.22 μm filter membrane was done for two turns. The solution was further filtered using a dialysis membrane against deionized water for 48 h. The resultant supernatant was stored at 4 °C for further use.

### Preparation of thin film

Quartz and FTO substrates were used in the studies for electrical measurements. In cleaning the substrates, firstly, the substrates were dipped in a solution of 2 ml of Hellmanex III and 200 ml of hot distilled water and sonicated for 10 min. The substrates were then rinsed thoroughly with distilled water. The substrates were further cleaned using an ozone cleaner for 10 min at a temperature of 24 °C. Thin film deposition of the few-layered graphene sheets was achieved by drop casting on the substrate and allowing it to air dry.

### Electrochemical measurements

Electrochemical measurements were performed on specially designed electrodes fabricated by the Laser-Induced Graphene (LIG) technique. The LIG is a graphene synthesis technique that involves exposing a polymeric precursor to laser light to cause photochemical and thermal conversion into graphene^29^. One significant benefit of LIG is high conductivity^30^, a required condition for substrate selection in making electrodes for electrochemical measurements. Therefore, as part of this work, we also aim to examine the practicality of LIG as conducting substrates.

LIG electrode patterning was achieved in one step on a Kapton paper by using a high-powered ${CO}_{2}$ laser engraving machine (Versa LASER, VLS 3.50) of 10.6 µm wavelength, a voltage of 220 V, and a power of 5 W.

200 μl of the synthesized graphene was then drop-casted on LIG, which functioned as the conducting substrate for our synthesized graphene. After drying at room temperature for 48 h, a gel electrolyte layer was deposited, and the sample was dried for 6 h. Gel electrolyte was made by dissolving 5 g of PVA powder in 5 ml of ${H}_{2}{SO}_{4}$ and 45 ml of deionized water at $90 ^\circ \mathrm{C}$ while stirring vigorously until a homogeneous solution was formed. After cooling, the solution was frozen at − 20 °C and thawed at 25 °C for 3 h to obtain the gel electrolyte. Measurements were made for LIG and the drop-casted sample on LIG (LIG + PG). PG refers to our synthesized graphene from pomegranate. The effectiveness of supercapacitors was then evaluated using Cyclic Voltammetry (CV), Galvanostatic Charge–Discharge (GCD), and Electrochemical Impedance Spectroscopy (EIS) with the VersaSTAT4 potentiostat.

### Characterization

The microstructure of the samples was studied using a high-resolution transmission electron microscope (TEM), JEM-2100F from JEOL Company, fortified with a 200 kV field emission gun. The elemental analysis was achieved by EDX, using an Oxford instrument 80 ${\mathrm{mm}}^{2}$ X-max detector system with point and ID mode. Raman Spectral analysis was conducted using the Micro Raman Confocal Microscope Witec Alpha300 model. Crystallinity and nature of synthesized GQDs were studied by an X-ray diffractometer 6100F from Shimadzu Company with a maximum power of 3 Kw, current of 30.0 mA, a divergent slit of 1.00 degree, scatter slit of 1.00, receiving slit of 0.3 mm with a scan range of 5–80 and a scan speed of 8 degrees per minute. The Fourier Transform Infrared spectra (FTIR) analysis was achieved using VERTEX 70v from Bruker Company with KBr as the reference. The optical structure was characterized by UV–Vis Spectroscopy using the Hitachi U-3900 UV spectrophotometer and a 10 mm path-length UV cell.

### Guideline statement

Experimental research and field studies on plants (either cultivated or wild), including the collection of plant material, must comply with relevant institutional, national, and international guidelines and legislation.

## Results and discussion

### Structural properties

**Figure 1:**
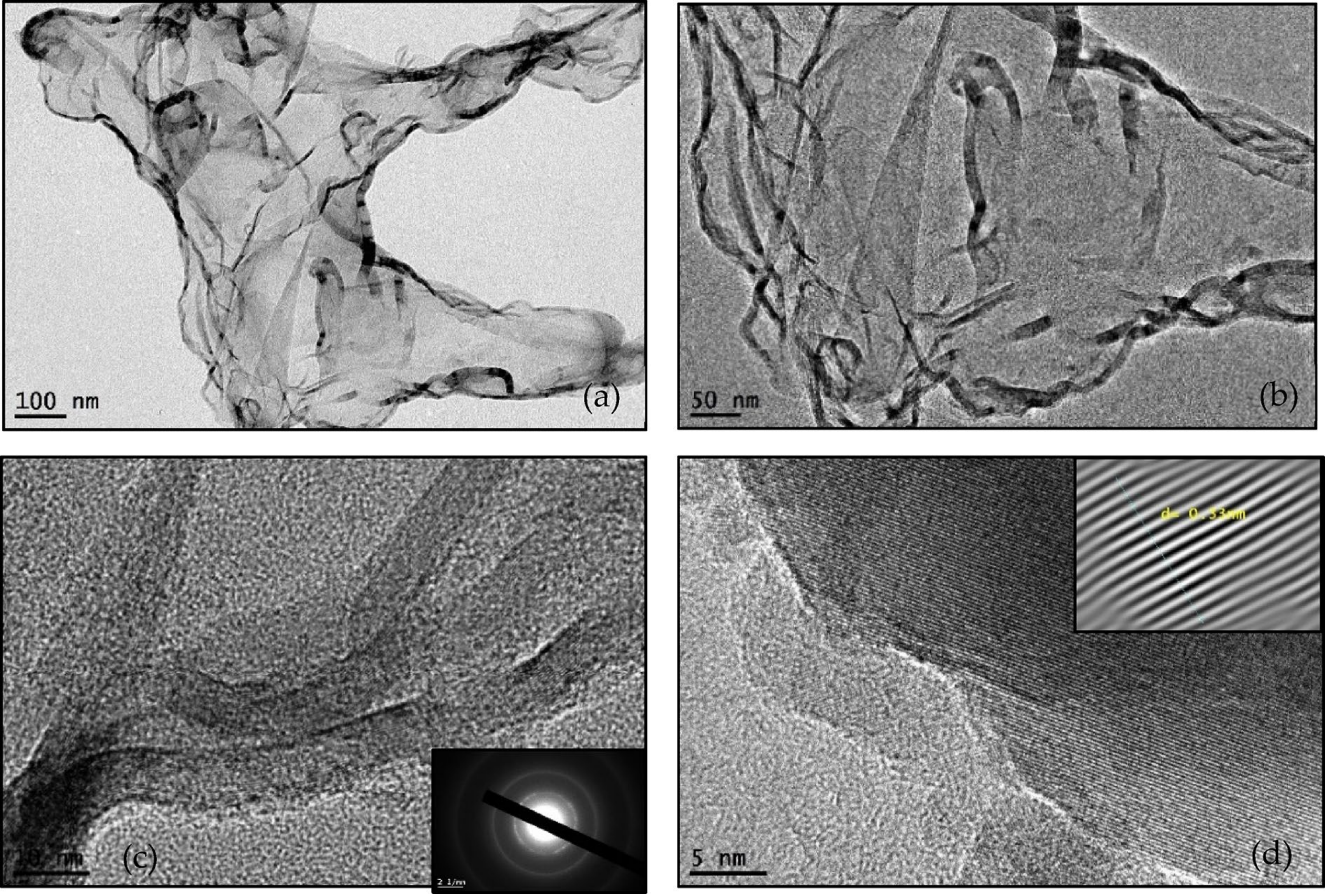
TEM micrographs of synthesized few-layered graphene sheets with different magnifications.

Figure 1a,b shows the TEM micrographs of the synthesized graphene at 80 °C with different magnifications. Based on the TEM analysis, the width of graphene lies in the range of ~ 100–400 nm with a lattice spacing of ~ 0.33 nm. Also, the inset of Fig. 1c shows a ring diffraction pattern, indicating some level of crystallinity of the synthesized graphene. High-resolution transmission electron microscope (HRTEM) images of the few-layered graphene sheets are shown in Fig. 1c,d. The HRTEM reveals the approximate number of layers of 2–4.

The elemental composition estimated by Energy dispersive X-Ray spectroscopy (EDX) is tabulated below. From Table 1, carbon is the majority, constituting 98.29% of the entire weight, and Oxygen, 1.71% weight. No extra elements apart from Carbon and Oxygen were observed, indicating high-quality synthesized graphene. The small number of oxygen molecules present could result from the oxidation of graphene due to its reaction with air.

**Table 1:** Results for EDX analysis of synthesized few-layered graphene sheets.

Element	Weight (%)	Atomic (%)
C	98.29	98.71
O	1.71	1.29

**Figure 2:**
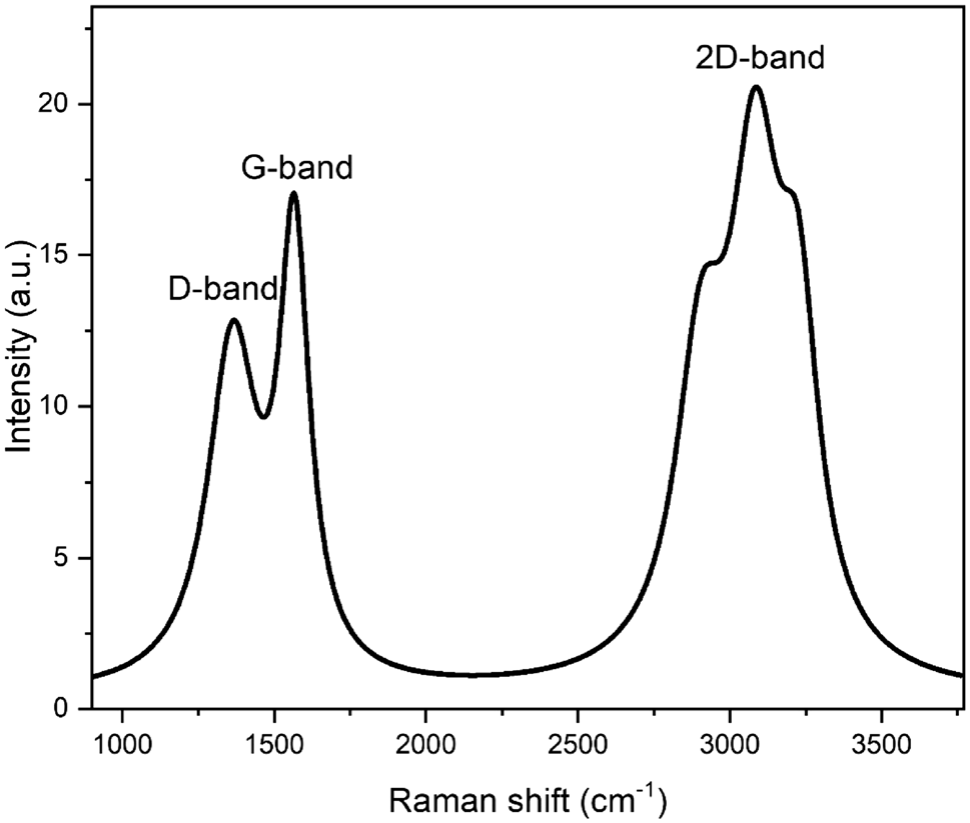
Raman spectra of synthesized few-layered graphene sheets.

Figure 2 shows the Raman spectra of the synthesized few-layered graphene sheets. Two peaks are observed at 1368.42 ${cm}^{-1}$ and 1564.42 ${cm}^{-1}$. These values correspond to the D and G bands, respectively. The observed D band is due to disordered planes, and its intensity is prominent in few-layered graphene compared to single-sheet graphene. The D and G bands' intensity ratio (R) was 0.73, suggesting the excellent quality of the obtained few-layered graphene sheets^31^. For the 2D band, three peaks are observed at ~ 2834.42, 3087.70 ${cm}^{-1}$ and 3199.02 ${cm}^{-1}$. The splitting of the 2D band into these three peaks results due to the double-resonance Raman process which involves phonon-electron scattering^21^. Also, the observed 2D band at 2834.42 cm^–1^ is blue-shifted due to the strain and vibrational phonons related to impurities^32^. On the other hand, the combinations of deconvoluted peaks at 3087.7 cm^-1^ and 3199.02 cm^-1^ can be ascribed to the D + G band^33^. The G to the 2D band ratio (i.e. I_G_/I_2D_) was also calculated to be 0.98, indicating layer numbers n < 5^34^, which is consistent with that obtained from the HRTEM images.

**Figure 3:**
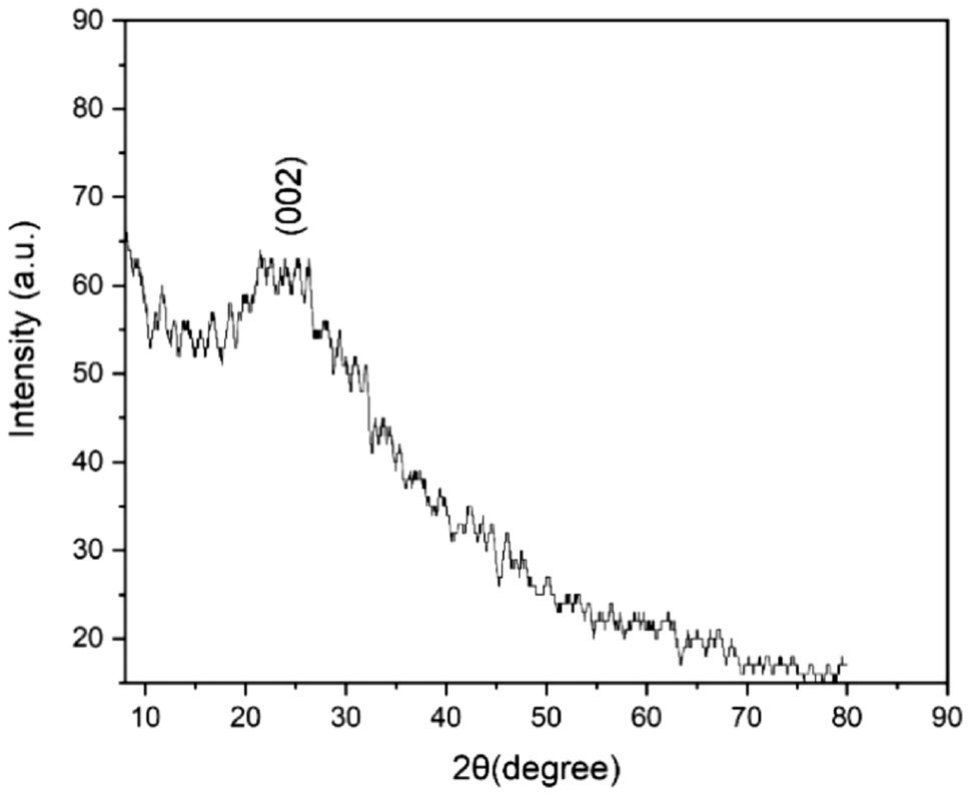
XRD spectrum of synthesized few-layered graphene sheets.

The XRD pattern of the synthesized few-layered graphene sheets is presented in Fig. 3. A broad diffraction peak centred at 2θ value of ~ 24° with a calculated d-spacing of 0.37 nm corresponding to the 002 planes of graphitic structures is observed^35^. The broad spectrum observed is due to the amorphous nature of the synthesized few-layered graphene sheets^36^, specifically, the disorder of graphite layers caused by empty rooms between graphite layers^37^. The broad peak is also observed due to the broadening of the d spacing^21^.

**Figure 4:**
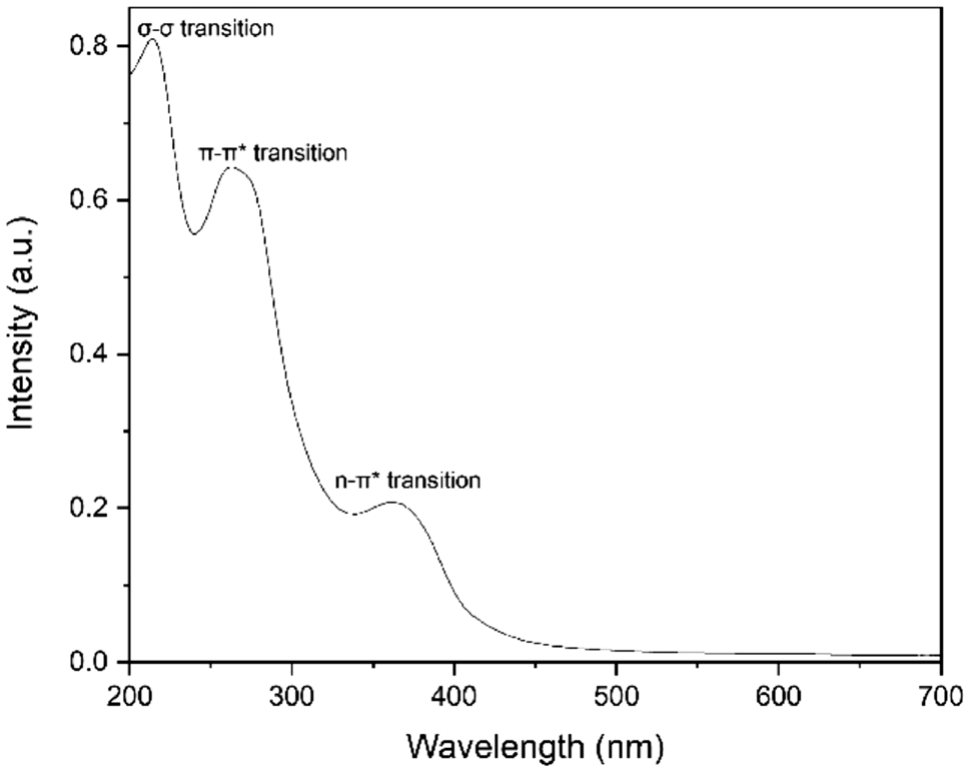
UV–visible spectrum of synthesized few-layered graphene sheets.

UV–vis spectra of the synthesized few-layered graphene were studied using the U-3900 spectrophotometer in the 200–800 nm wavelength range. As shown in Fig. 4, three peaks were observed at 214 nm, 256 nm, and 364 nm. The peak observed at 214 nm is assigned to sigma-sigma transitions, while the peak at 256 can be attributed to π-π* electronic transitions of s ${p}^{2}$ C=C bonds of the aromatic rings^7,38^, and the peak located at 364 nm is attributed to the n-π* transitions of the fewer C=O bonds present.

**Figure 5:**
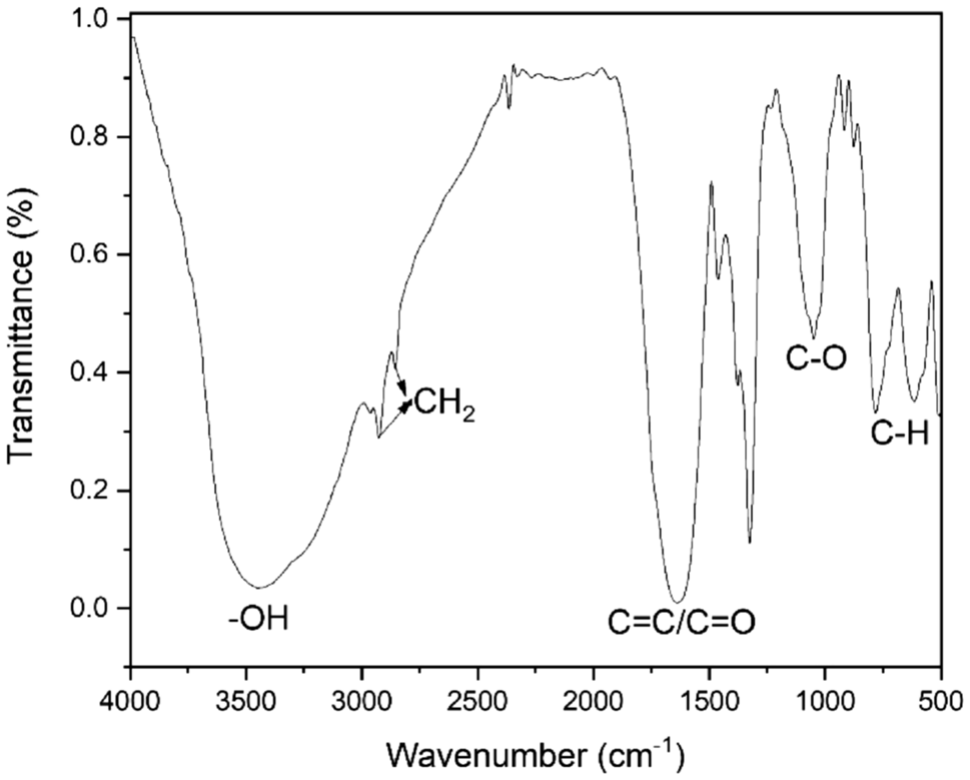
FTIR spectra of synthesized few-layered graphene sheets.

Figure 5 shows the FTIR spectra of the synthesized few-layered graphene sheets. From the spectra, a broad band is seen at 3,440.44 ${cm}^{-1}$ which is due to the stretching vibration of the *–OH* groups and the absorbed water molecules. The peaks observed at 2925.10 ${cm}^{-1}$ and 2853.79 ${cm}^{-1}$ arise from $-{CH}_{2}$ stretching vibrations. The intense peak at 1637.31 ${cm}^{-1}$ is a result of the combination of *COOH* group stretching vibration and *C*=*C* skeletal vibrations from graphitic domains in the ketone or quinone groups. The weak peak observed at 1380.91 ${cm}^{-1}$ is due to the presence of *C–O,* and lastly, the observed peaks between 721.996 and 570.56 ${cm}^{-1}$ correspond to the bending of *C-H*^39–41^.

Oxygen functional groups on the surface of the synthesized few-layered graphene sheets make it hydrophilic, explaining its solubility in water.

### Electrical properties

**Figure 6:**
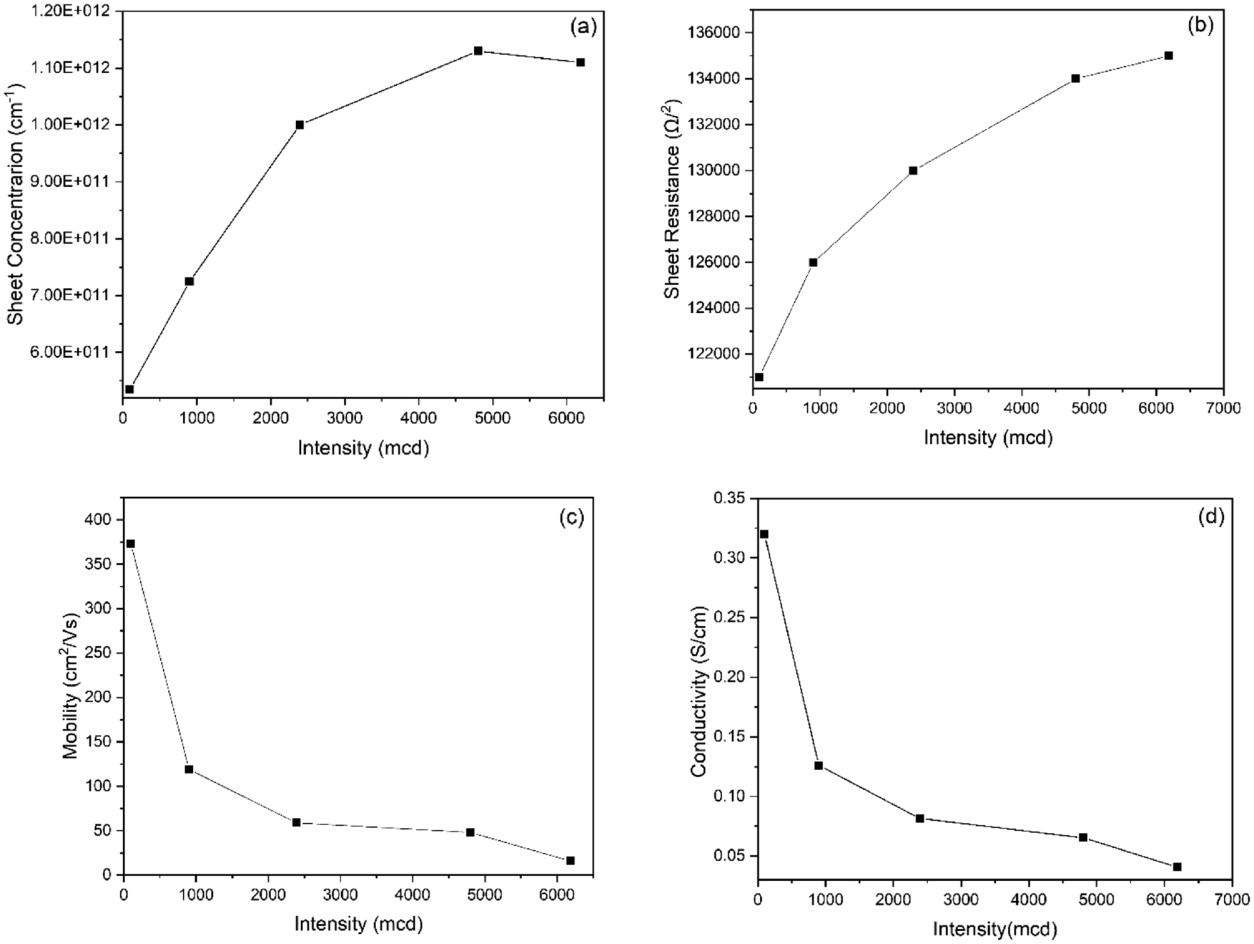
Light intensity versus (**a**) sheet concentration, (**b**) Sheet resistance, (**c**) Mobility, and (**d**) Conductivity of synthesized few-layered graphene sheets.

To examine the electrical properties of the synthesized few-layered graphene sheets, Hall Effect measurements were conducted. Figure 6a–d shows the behavior of the few-layered graphene sheets under white light as the light intensity increases. Generally, the sheet concentration and sheet resistance increases with increasing intensity. In contrast, the electron mobility and conductivity decrease with increasing light intensity, indicating that our synthesized graphene sheets are light-sensitive and can find potential applications in optoelectronics.

### Plausible mechanism for graphene formation

**Figure 7:**
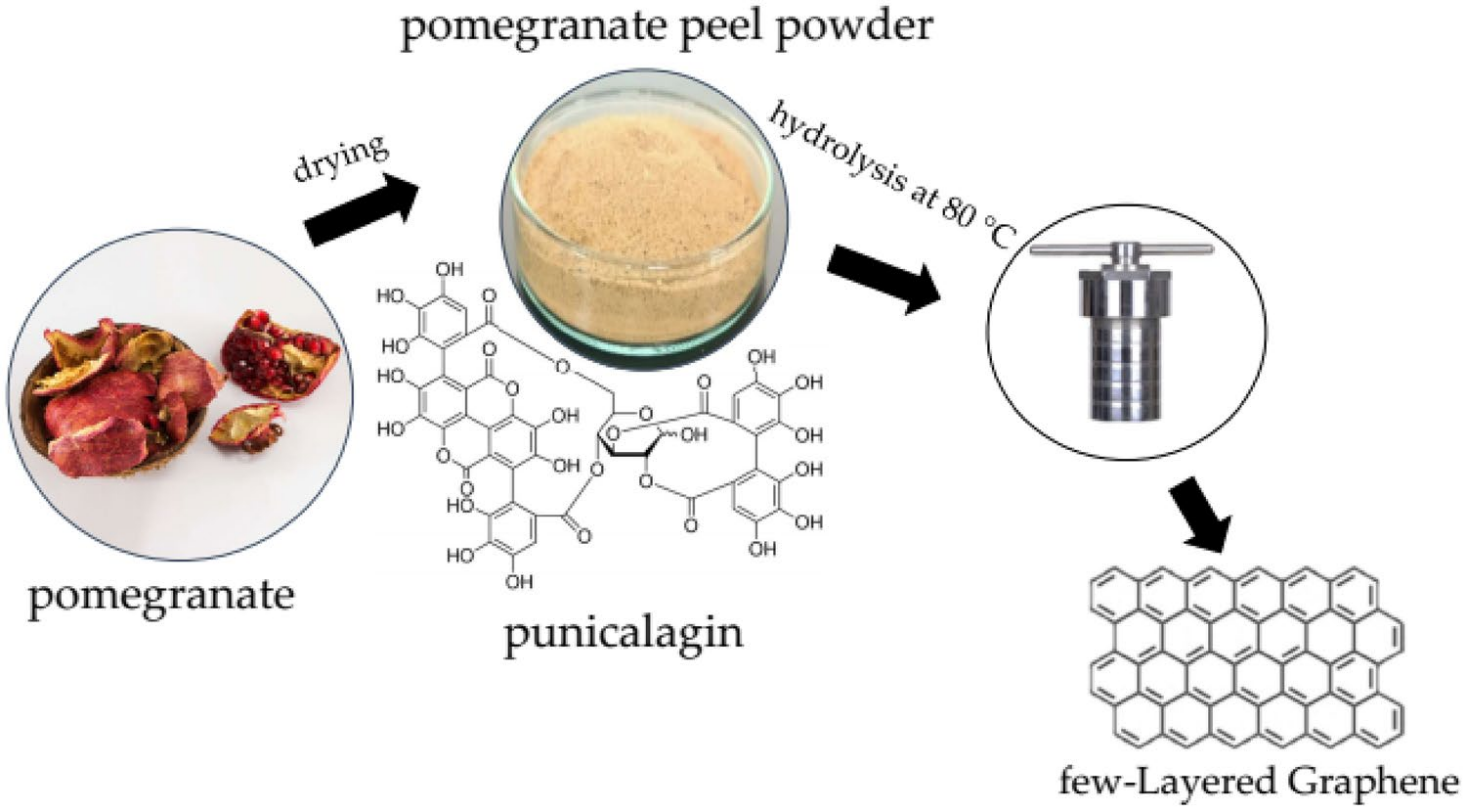
Schematics for forming few-layered graphene sheets from pomegranate peel.

The pomegranate peels consist mainly of hydrolyzable punicalagin, a bioactive compound with a chemical structure including gallagic acid and ellagic acid connected through a glucose molecule. This compound has a common feature, the presence of labile oxygen linkages, responsible for the cyclization and metamorphosis of the punicalagin molecules. Most likely, the heating temperature at 80 °C and the pressure inside the autoclave are capable of hydrolyzing punicalagin and breaking these labile oxygen linkages resulting in the release of ellagic acid. Therefore the reaction proceeds by the breaking of the labile bonds, followed by the release of ${H}_{2}O$ and then the subsequent aromatization leading eventually to the formation of few-layered graphene sheets. The schematic diagram for few-layered graphene sheet formation is shown in Fig. 7.

### Electrochemical analysis

**Figure 8:**
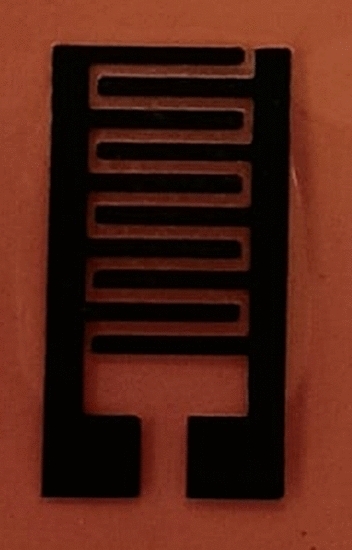
A digital photograph of LIG electrode pattern on Kapton paper.

**Figure 9:**
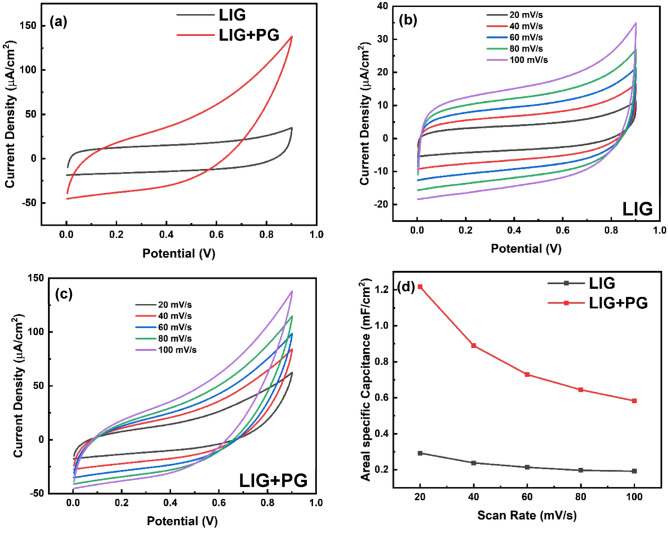
(**a**) Comparative CV profiles of LIG and LIG + PG at 20 mV ${s}^{-1}$. CV at different scan rates for (**b**) LIG and (**c**) LIG + PG. (**d**) Change in areal specific capacitance at different scan rates for LIG and LIG + PG.

Cyclic voltammetry was performed for the LIG Fig. 8 and the synthesized few-layered graphene (PG) on LIG substrate (LIG + PG). The behavior of both electrodes was investigated within the same potential window of (0–0.9)V and at scan rates in the range of 10–100 m ${Vs}^{-1}$. As shown in Fig. 9a, the obtained curves for both samples show rectangular curves, which are typical of an Electric Double Layer Capacitor (EDCL)^42^. Rectangular-shaped CV curve also indicates low contact resistance and ideal charge propagation of the electrodes^43^.

Comparatively, LIG + PG has a larger CV area, indicating higher capacitance and better rate capability. Also, from Fig. 9b and c, it can be seen that the curves of both samples are more rectangular at lower scan rates than that at higher scan rates. This is because higher scan rates usually generate considerable Ohmic resistance, effectively distorting the CV loop and narrowing the loop with an oblique angle^44^. A plot of the Capacitance versus the Scan rates is shown in Fig. 9d. From the graph, it is evident that the capacitance of both samples decreases as the scanning rate increases. This result is expected of carbon-based materials because of the limited transfer of ions to the carbon particle surface, which results in areas of the electrode layer not being accessible at high scan rates^43^. Also, at low scan rates, a more homogeneous potential with minimal variations is created along the material^45^. The specific areal capacitance at 20 m ${Vs}^{-1}$ for LIG is 0.29 mF ${cm}^{-2}$ and LIG + PG is 1.22 mF ${cm}^{-2}$ with the effective specific areal capacitance, i.e. the specific areal capacitance from our synthesized graphene, being 0.93 mF ${cm}^{-2}$ which is comparatively higher than some reported works^46^.

**Figure 10:**
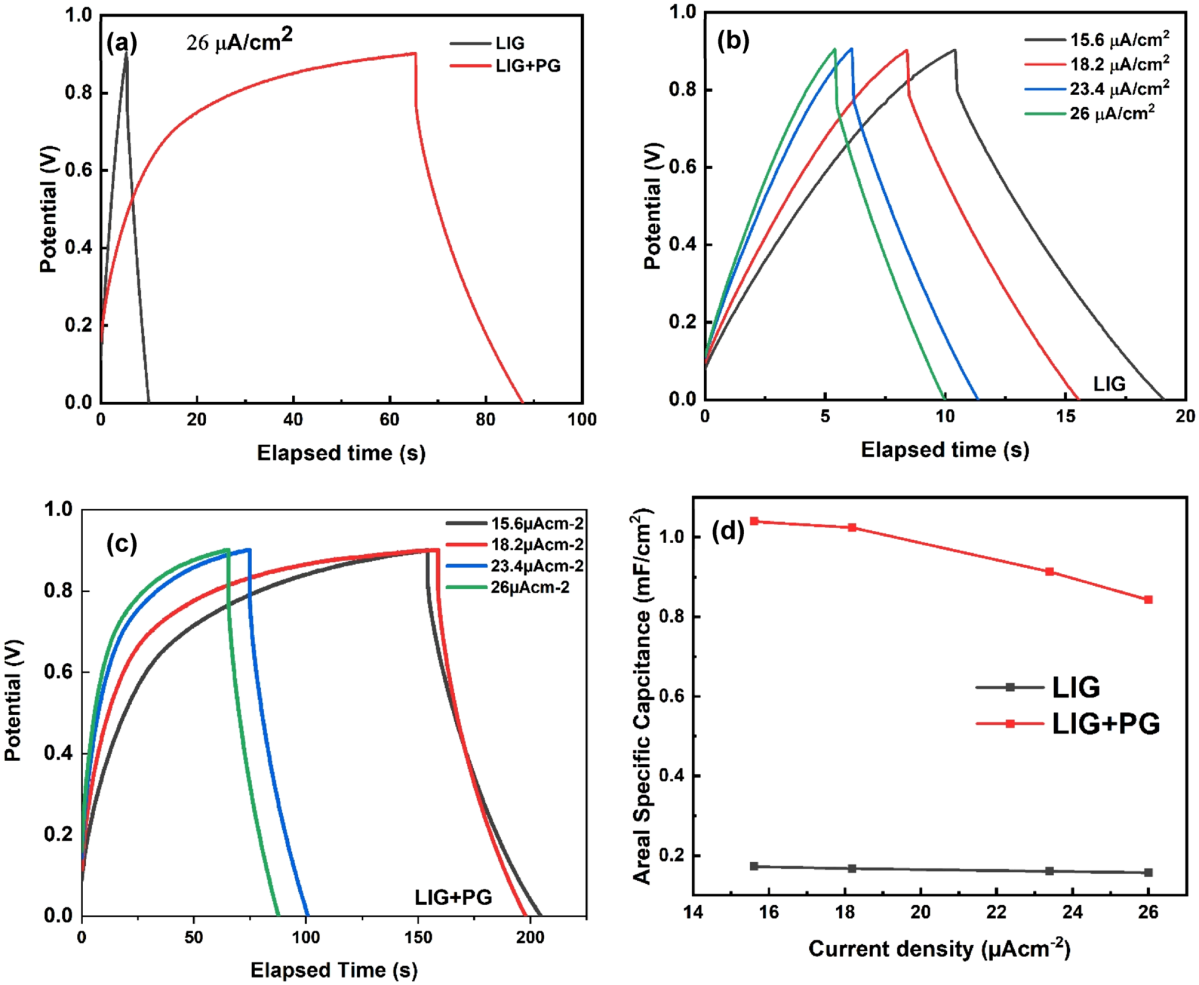
(**a**) Comparative GCD curves for LIG (in red) and LIG + PG (in black). (**b**) GCD curves for LIG at different current densities (**c**) GCD curves for LIG + PG at different current densities (**d**) Areal specific capacitance versus current density of both samples: LIG (in red), LIG + PG (in black).

The GCD behavior of the samples was studied at various current densities within the range of 10–50 μA ${cm}^{-2}$ as shown in Fig. 10b and c. Generally, as the current density increases, the effective area decreases. Figure 10a shows the comparative GCD curve at a current density of 26 μA ${cm}^{-2}.$ As seen, the LIG + PG exhibits a longer charging and discharge than the LIG, and this is an indication of higher capacitance for the former.

A sharp voltage drop is observed at the beginning of discharge for both samples, which is due to the diffusion-limited mobility of the electrolyte ions in the electrode pores and could result from the interface resistance between the current collector and the active layer or the conductivity of highly porous activated carbon^45^. Voltage drop for LIG was found to be 0.11 V, and that of LIG + PG was found to be 0.082 V, with the difference equating to 0.028 V. This indicates that our synthesized few-layered graphene sheets contributed a relatively small amount, almost negligible, to the voltage drop.

A plot of the areal specific capacitance versus current density is shown in Fig. 10d. The areal specific capacitance values decrease as the current densities increase. The equation ${C}_{A}$ = $\frac{I\Delta t }{{A}_{act}\Delta V}$, was used in calculating the areal specific capacitance (areal) for both samples at different current densities. As expected, the LIG + PG had relatively high areal specific capacitance values. At 15.6 μA ${cm}^{-2},$ the areal specific capacitance for LIG and LIG + PG was 0.66 mF ${cm}^{-2}$ and 3.99 mF ${cm}^{-2}$ respectively with the effective capacitance having a value of 3.39 mF ${cm}^{-2}$, comparatively higher than previously reported works^47^ and thus indicating good capacitor behavior of our synthesized graphene. A summary of the electrochemical performance of the sample can be found in Table 2.

**Table 2:** Summary of electrochemical parameters for the fabricated cells.

Parameter	LIG	LIG + PG	PG
${C}_{A}$, mF ${cm}^{-2}$	0.66	3.99	3.33
E, mWh ${cm}^{-2}$	1.5 $\times {10}^{-5}$	9.66 $\times {10}^{-5}$	8.16 $\times {10}^{-5}$
P, mW ${cm}^{-2}$	0.006	0.007	0.001
ESR, Ω	3.53	11.98	8.42
iR drop, V	0.11	0.082	0.028

The areal specific capacitance, energy density, and maximum power density were calculated with GCD measurements at a current density of 15.6 μA ${cm}^{-2}.$

### Electrochemical impedance spectroscopy (EIS)

**Figure 11:**
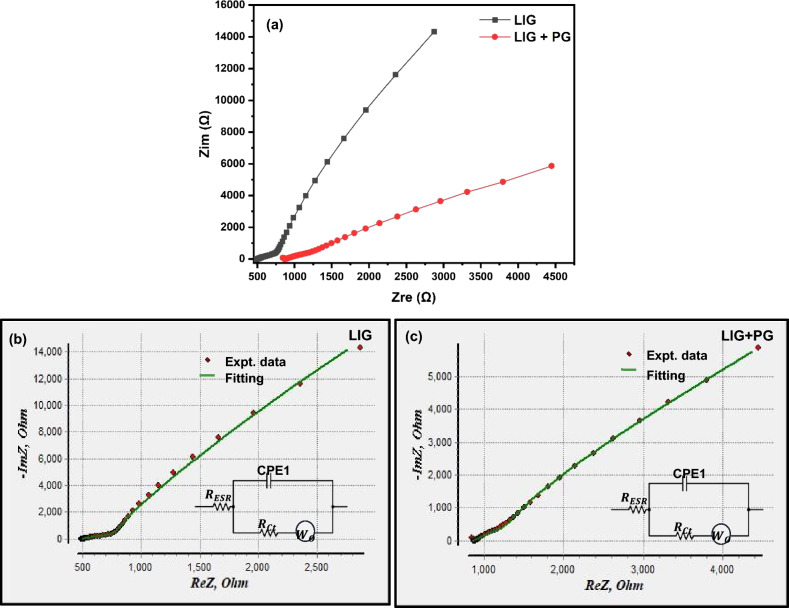
(**a**) Nyquist plot of LIG (in black) and LIG + PG (in red) in the frequency range 100 MHz–0.1 Hz (**b**) Nyquist plot with fitting and an equivalent circuit model (inset) of LIG (**c**) Nyquist plot with fitting and an equivalent circuit model (inset) of LIG + PG.

EIS was further used to characterize the performance of the graphene samples. The experiment was performed in the frequency range of 100 MHz to 0.1 Hz. Figure 11a shows the Nyquist plots obtained from the EIS data. The Nyquist plots obtained consists of a straight line segment with a slope of 45° followed by another line with a steeper slope as expected of electric double-layer capacitors. Also, the absence of a semi-circle further confirms the EDCL behavior^42^, corroborating the results obtained in the CV measurements.

EIS plot fitting was done using the EIS Spectrum Analyzer to get the equivalent circuit model of the EIS plot, shown in Fig. 11b and c. The first part of the equivalent circuit gives information about the equivalent series resistance ($${R}_{ESR})$$. $${R}_{ESR}$$ for LIG was found to be 495.17 Ω and that of LIG + PG was found to be 859.47 Ω. Additionally, the second component of the circuit is a parallel assembly of the constant phase element (CPE 1), Warburg impedance (Wo), and charge transfer resistance (Rct), which represents the charge transfer phenomenon of porous graphene electrodes^46^. The charge transfer resistance ($${R}_{ct})$$ for LIG was 25.61 Ω and that of LIG + PG was 33.09 Ω. The lower values of $${R}_{ct}$$ signifies lower ionic transfer resistance effect within the smaller pores inside the electrode active sites^46^. Our synthesized graphene's relatively lower resistant values make it an excellent choice for energy storage since lower resistant values are necessary for minimizing supercapacitor losses and increasing overall efficiency.

## Conclusion

In summary, we have successfully developed for the first time a green, facile, and energy-efficient method of synthesizing few-layered graphene sheet from pomegranate peels via the hydrothermal method at a low temperature of 80 °C without the aid of any passivating agent. The microstructure, morphology, and electrical properties studies show that our synthesized graphene is of high quality. Excellent electrochemical performance has also been achieved with the areal capacitance of 3.39 mF ${cm}^{-2}$ at a current density of 15.6 μA ${cm}^{-2}$, making our synthesized graphene applicable as a supercapacitor for energy storage.

## Data Availability

The datasets used and/or analyzed during the current study are available from the corresponding author upon reasonable request.
